# Cell viability measured by cytotoxicity assay as a biomarker of chronic obstructive pulmonary disease exacerbation: a prospective cohort study

**DOI:** 10.1038/s41598-025-14536-5

**Published:** 2025-08-07

**Authors:** Ye Jin Lee, Eun-Young Eo, Dong Hyun Joo, Si-mong Yoon, Hyung-Jun Kim, Myung Jin Song, Byoung Soo Kwon, Yeon Wook Kim, Sung Yoon Lim, Yeon-Joo Lee, Jong Sun Park, Young-Jae Cho, Jae Ho Lee

**Affiliations:** 1 Division of Pulmonary and Critical Care Medicine, Department of Internal Medicine, Seoul National University Bundang Hospital, Seongnam, Republic of Korea; 2 Department of Internal Medicine, Seoul National University College of Medicine, Seoul, Republic of Korea; 3 Divisions of Pulmonary and Critical Care Medicine, Department of Internal Medicine, Seoul National University College of Medicine, Seoul National University Bundang Hospital, Seongnam, Republic of Korea; 4 Division of Pulmonary and Critical Care Medicine, Department of Internal Medicine, Seoul National University Bundang Hospital, 82 Gumi-ro 173beon-gil Bundang-gu, Seongnam-si, Gyeonggi-do Republic of Korea

**Keywords:** Cell viability, Chronic obstructive pulmonary disease, Exacerbation, Lactate dehydrogenase cytotoxicity assay, Cell biology, Medical research, Risk factors

## Abstract

Acute severe exacerbation of chronic obstructive pulmonary disease (COPD) is related to high mortality; however, a robust blood biomarker for COPD exacerbation has not been established. Impaired clearance of apoptotic cells is a possible pathogenesis of COPD development. We evaluated the clinical utility of serum cell viability as a predictive biomarker for COPD exacerbation.Using serum from patients with stable COPD, cell viability was analyzed with a lactate dehydrogenase (LDH) assay. The patients were divided into low (optical density [OD] > 0.737) and high (OD ≤ 0.737) cell viability groups. Poisson regression analyses estimated the prognostic impact for COPD exacerbation, and a Cox proportional hazard model determined the impact on mortality. Among 162 patients, 47 were excluded due to follow-up loss within 1 year, asthma or combined interstitial lung disease diagnosis, and unsuitable cell viability measurements. The median follow-up duration was 6.3 years (range 0.7–11 years); 61 (53%) patients experienced at least one moderate or severe exacerbation, and 21 (19.7%) died. Patients in the low cell viability group were older, more likely to have poor quality of life and had a lower proportion of the non-exacerbator phenotype than those in the high cell viability group. The low cell viability group had a higher risk of moderate (incidence rate ratio [IRR], 1.58; *p* = 0.049) and severe (IRR, 2.69; *p* = 0.001) exacerbations and mortality (adjusted hazard ratio, 5.79; *p* = 0.016).We identified that low cell viability, measured with a serum LDH cytotoxicity assay, was associated with severe COPD exacerbation and higher mortality in patients with COPD.

**Supplementary Information:**

The online version contains supplementary material available at 10.1038/s41598-025-14536-5.

## Introduction

Chronic obstructive pulmonary disease (COPD) is the eighth leading cause of years of life lost globally, and acute exacerbation is associated with deterioration of lung function, reduced quality of life, and economic losses^1,2^. In a 5-year follow-up study, Waeijen-Smit et al. reported that the mortality rate for patients with severe COPD exacerbation requiring hospitalization to be up to 58.2%^3^. Hence, it is important to identify predictive biomarkers to determine the individual predisposition to severe acute COPD exacerbations. Several predictive models for COPD exacerbation have been proposed, which are based on factors including prior exacerbation history, forced expiratory volume in 1 s (FEV_1_), COPD Assessment Test (CAT) score, air pollution data, epidemic respiratory virus, medication use, comorbidities, eosinophil count, and smoking status. Although the ability of these models to predict COPD exacerbation is high, considering too many factors in the model presents disadvantages of being difficult to use and time consuming in real-world clinical practice^4,5^.

Previous research has utilized single blood biomarkers to predict COPD exacerbation, most notably C-reactive protein (CRP), interleukin-6, eosinophil count, and tumor necrosis factor-α^6,7^. These biomarkers, particularly CRP and eosinophils, are widely used in clinical practice due to their ease of accessibility and utility in evaluating inflammation in respiratory disease. Among them, C-reactive protein has been the most commonly evaluated biomarker in previous studies. Its levels are elevated during acute exacerbation of COPD (AECOPD) compared to those during stable COPD; however, these data derived only from retrospective studies or case-control study while prospective studies were limited, with CRP could not significantly predict AECOPD at longer follow up study or study with older patients^8,9^. Additionally, blood eosinophils offer promising biomarker for evaluating inhaled corticosteroid (ICS) responses^10,11^. However, their potential as a predictive biomarker for future COPD exacerbation is controversial^12–14^.

Impaired clearance of apoptotic cells may result in COPD development^15,16^^17^. Asare et al. discovered that the exposure of cigarette smoking reduce the expression of RUN domain Beclin-1-interacting and cysteine-rich domain-containing protein (Rubicon), a scaffold protein essential for a critical process in phagolysosomal function during efferocytosis. Reduced Rubicon levels are associated with dysregulated LC3-associated phagocytosis (LAP), which is indispensable pathway for effective efferocytosis^18,19^. This impaired efferocytosis has been related with sustained inflammation in chronic respiratory diseases^20^. Another study using sputum of patients of COPD showed that impaired eosinophil clearance by macrophages was associated with the frequency of COPD exacerbation. However, this finding has been demonstrated only in an in vivo model or, using sputum, or airway epithelial cells in lung tissue^17^. Additionally, no studies have suggested an effect of the presence of more apoptotic cells within serum following COPD exacerbation or the detection of dead cells within serum of patients with COPD. This study aimed to determine the predictive value of serum cell viability measured using a lactate dehydrogenase (LDH) cytotoxicity assay for acute exacerbation of COPD.

## Results

### Patient demographics

**Fig. 1:**
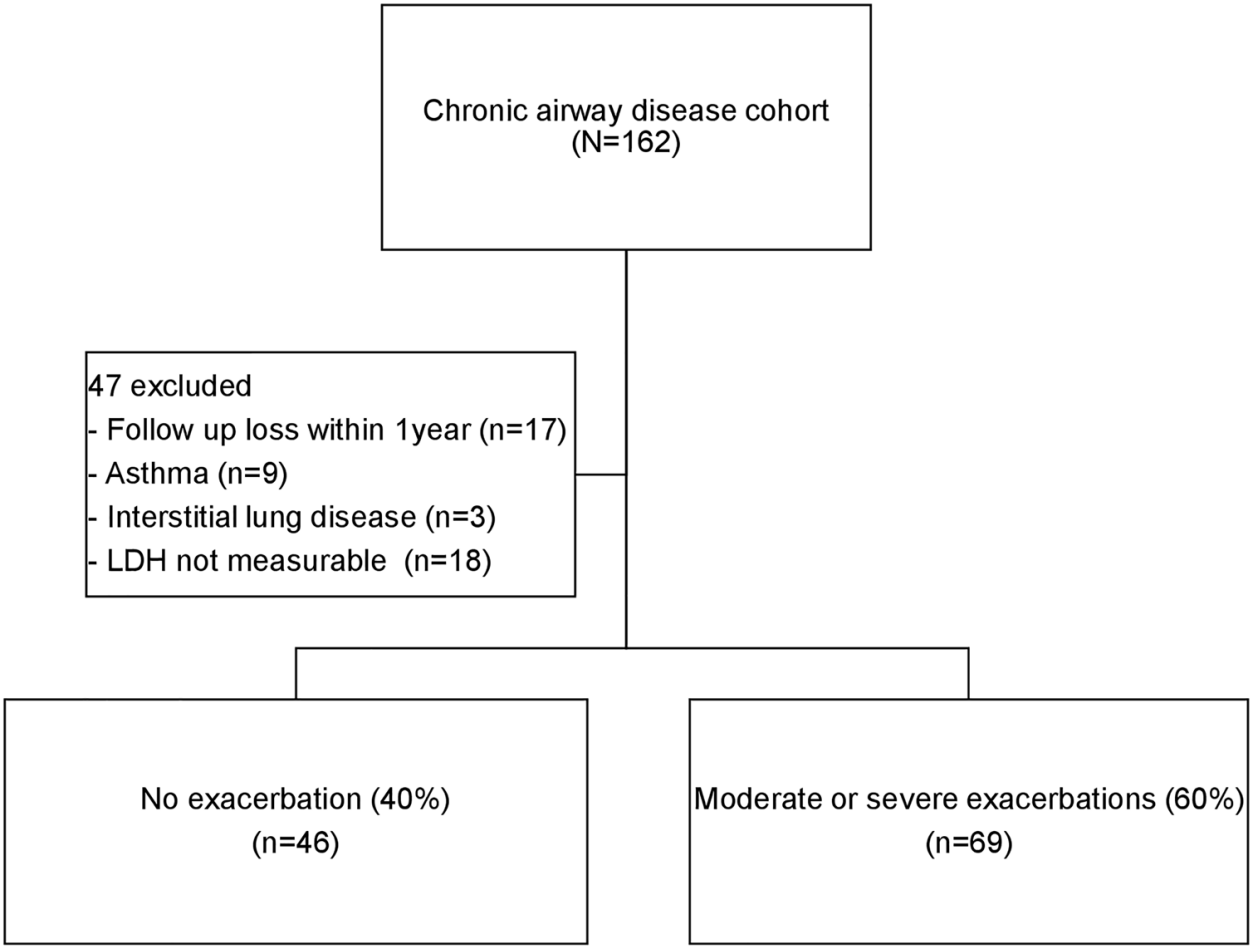
Study flow chart.

Overall, 162 patients were enrolled in the chronic airway prospective cohort, and 115 were analyzed after the exclusion of 47 patients for the following reasons: inadequate cytotoxicity assay data (due to failure to accurately measure LDH level caused by excessive cell lysis: 10 patients, and insufficient sample to analyze after rewarming: 8 patients), follow-up loss within 1 year (4 patients were transferred, 13 patients mostly had mild symptoms [median mmRC score: 1] and preserved lung function [FEV1 2.2 ± 0.8 L]) : 17 patients, diagnosis of asthma: 9 patients, and diagnosis of interstitial lung disease with chronic airway disease: 3 patients (Fig. 1). The baseline characteristics are described in Table 1. The median follow period of this cohort was 6.3 years (range 0.7–11 years); 61 (53%) patients experienced at least one severe exacerbation within one year prior to enrollment, and 21 (18.8%) died during follow-up. The mean age was 70.2 ± 8.0 years, most patients were male (95.6%), only 4 (3.5%) had never smoked (three had post-tuberculosis bronchiectasis and one had uncontrolled asthma that progressed to fixed air flow limitation consistent with COPD), and the median cell viability value was 0.737 (P25-P75, OD: 0.65–0.84). We divided patients into low cell viability (OD > 0.737) and high cell viability (OD ≤ 0.737) groups according to the median cell viability value. The low cell viability group was older (71.8 ± 7.9 vs. 68.7 ± 7.8, *p* = 0.038) and more likely to have poor quality of life in the activity (53.8 ± 20.0 vs. 40.2 ± 19.0, *p* < 0.001) and impact (25.9 ± 18.1vs 16.4 ± 16.1, *p* = 0.004) domains of the SGRQ relative to the high cell viability group. There were no significant between-group differences regarding body mass index (BMI), smoking status, comorbidities, type of inhaler used, and lung function. Patients in the low cell viability group showed a trend towards experiencing more severe exacerbation in the previous year, were likely to walk approximately 34 m less in the 6-min walk test, and had higher levels of blood eosinophils than those in the high cell viability group; however, these differences failed to show statistical significance. Among the total population, 46 (40%) patients were classified as the non-exacerbator phenotype, and the proportion of this phenotype was higher in the high cell viability group (47.0%) than in the low cell viability group (33.3%) (*p* = 0.005). Additionally, mortality was significantly higher in the low cell viability group (28.1%) than in the high cell viability group (9.1%) (*p* = 0.025).

**Table 1:** Baseline characteristics of enrolled patients.

	High cell viability (OD ≤ 0.737)(*n* = 58)	Low cell viability (OD > 0.737) (*n* = 57)	Total (*n* = 115)	^*^P value
Age	68.7 ± 7.8	71.8 ± 7.9	70.2 ± 8.0	0.038
Male, n(%)	56 (96.5%)	54 (94.7%)	110 (95.6%)	0.679
BMI	21.7 ± 3.5	21.7 ± 4.0	21.7 ± 3.8	0.947
Smoking status	-	-	-	0.314
Never smoker, n(%)	3 (5.2%)	1 (1.8%)	4 (3.5%)	-
Ever smoker, n(%)	**40 (68.9%)**	**46 (80.7%)**	86 (74.8%)	-
Current smoker, n(%)	15 (25.9%)	10 (17.5%)	25 (21.7%)	-
Pack year	43.2 ± 19.8	48.4 ± 25.6	45.6 ± 22.7	0.262
Severe exacerbation history in previous year, n(%)	7 (12.3%)	12 (20.7%)	19 (16.5%)	0.316
CAT score	12 [10–18]	15 [10–20]	13 [10–19]	0.303
6MIN WT	434.7 ± 101.4	400.1 ± 122.9	417.6 ± 113.4	0.111
SGRQ	-	-	-	-
Symptom	40.5 ± 18.1	45.3 ± 17.0	43.0 ± 17.6	0.159
Activity	40.2 ± 19.0	53.8 ± 20.0	47.1 ± 20.6	< 0.001
Impact	16.4 ± 16.1	25.9 ± 18.1	21.2 ± 17.7	0.004
Total	28.3 ± 14.9	37.2 ± 17.1	32.8 ± 16.6	0.004
Comorbidity, n(%)	-	-	-	-
DM	6 (10.3%)	8 (14.0%)	14 (12.2%)	0.581
Angina	9 (15.8%)	10 (17.2%)	19 (16.5%)	> 0.99
Heart failure	5 (8.7%)	4 (6.9%)	9 (7.8%)	0.743
Arrythymia	3 (5.3%)	5 (8.6%)	8 (7.0%)	0.717
Bronchiectasis	4 (7.0%)	2 (3.5%)	6 (5.2%)	0.438
Malignancy	5 (8.8%)	10 (17.2%)	15 (13.0%)	0.268
Inhaler	-	-	-	-
LAMA	22 (37.9%)	16 (28.1%)	38 (33.0%)	**0.323**
LABA + LAMA	9 (15.5%)	9 (15.8%)	18 (15.7%)	**> 0.999**
ICS + LABA	9 (15.5%)	12 (21.1%)	21 (18.3%)	**0.478**
ICS + LABA and LAMA	17 (29.3%)	17 (29.8%)	34 (29.6%)	**> 0.999**
Others	1 (1.7%)	3 (5.6%)	4 (3.5%)	**0.364**
Blood eosinophil (cells/Ul)	197.0 ± 162.4	270.4 ± 404.1	234.0 ± 309.8	0.206
**Post-bronchodilator FVC-liters**	3.2 ± 0.6	3.0 ± 0.6	3.1 ± 0.6	0.169
**Post-bronchodilator FVC-% of predicted value**	88.1 ± 13.1	86.1 ± 14.1	87.1 ± 13.6	0.436
**Post-bronchodilator FEV**_**1**_ **-liters**	1.5 ± 0.5	1.4 ± 0.5	1.5 ± 0.5	0.448
**Post-bronchodilator FEV**_1_ **-% of predicted value**	60.9 ± 19.0	60.3 ± 18.6	60.6 ± 18.7	0.859
**FEV1 ≥ 80% predicted**	**11 (18.9%)**	**6 (10.5%)**	**17 (14.8%)**	**0.585**
**50% ≤ FEV1 < 80% predicted**	**31 (53.5%)**	**33 (57.8%)**	**64 (55.7%)**	
**30% ≤ FEV1 < 50% predicted**	**13 (22.4%)**	**16 (28.1%)**	**29 (25.2%)**	
**FEV1 < 30% predicted**	**3 (5.2%)**	**2 (3.5%)**	**5 (4.3%)**	
**Post-bronchodilator ratio of FEV**_**1**_ **to FVC-%**	47.0 ± 11.9	47.0 ± 11.8	47.0 ± 11.8	0.999
0.6 ≤ ratio < 0.7	11 (19.0%)	9 (15.8%)	20 (17.4%)	0.241
0.5 ≤ ratio < 0.6	8 (13.8%)	16 (28.1%)	24 (20.9%)	-
0.4 ≤ ratio < 0.5	21 (36.2%)	14 (24.6%)	35 (30.4%)	-
Ratio < 0.4	18 (31.0%)	18 (31.6%)	36 (31.3%)	-
DLCO (%)	86.0 ± 24.5	82.1 ± 23.0	84.1 ± 23.7	0.375
TLC (%)	110.8 ± 17.2	108.4 ± 16.9	109.6 ± 17.0	0.473
RV (%)	122.1 ± 42.9	119.4 ± 44.1	120.8 ± 43.3	0.747
Non-exacerbator phenotype (%)	35 (60.3%)	19 (33.3%)	54 (47.0%)	0.005
Mortality	5 (9.1%)	16 (28.1%)	21 (18.8%)	0.025

Abbreviations. OD = optical density; BMI = body mass index; CAT, COPD Assessment Test; 6MIN WT = 6 min walk test; SGRQ = St George’s Respiratory Questionnaire; DM = diabetes mellitus; LAMA = long-acting muscarinic antagonists; LABA = long-acting β2 agonist; ICS = inhaled corticosteroids; FVC = forced vital capacity; _FE1V_= forced expiratory volume in 1 s; DLCO = diffusing capacity of the lungs for carbon monoxide; TLC = total lung capacity; RV = residual volume.

^*^P-value is for comparison between high cell viability and low cell viability group.

### COPD exacerbation rate

**Fig. 2:**
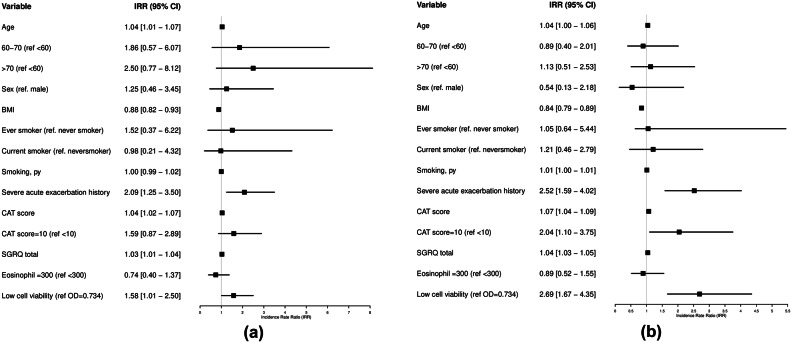
Forest plot of the incidence rate ratio for COPD exacerbation **a.** Forest plot of incidence rate ratio (95% CI) for moderate COPD exacerbation by risk factors **b.** Forest plot of incidence rate ratio (95% CI) for severe COPD exacerbation by risk factors.

Overall, 27 (47.4%) patients in the low cell viability group and 14 (24.1%) in the high cell viability group experienced at least one severe exacerbation (*p* = 0.01) during the follow-up period. The prospective annual moderate and severe exacerbation rate was 0.056 and 0.055 person-year, respectively. Compared with the high cell viability group, patients in the low cell viability group had a moderate exacerbation incidence rate ratio (IRR) of 1.56 (95% confidence interval [CI]: 0.98–2.50) and severe exacerbation IRR of 2.69 (95% CI: 1.66–4.35) (Fig. 2). We found that the moderate and severe exacerbation IRRs were similar between groups based on the eosinophil count (< 300 cells/µL and ≥ 300 cells/µL). A history of severe exacerbation within the previous year was a robust predictor of the annual moderate or severe exacerbation IRR (2.09 and 2.52, respectively). Older age, low BMI, high baseline SGRQ score, shorter 6-min walk test duration, and lower post-bronchodilator FEV_1_ and ratio of FEV_1_ to forced vital capacity (FVC) were associated with moderate exacerbations. Additionally, the incidence of severe exacerbation was significantly higher in patients with lower cell viability level, greater tobacco use, and higher baseline CAT score. Based on these outcomes, multivariate Poisson regression analyses showed that cell viability, smoking status, and baseline CAT score remained associated with a higher severe exacerbation incidence after adjustment for confounding variables. In particular, low cell viability was associated with a 2.21-fold higher incidence of severe exacerbation; patients with low BMI had a 1.13-fold higher incidence of moderate exacerbation (Table 2).

**Table 2:** Incidence rate ratio for moderate and severe exacerbation by risks.

	Moderate exacerbations		Severe exacerbations	
	Unadjusted IRR (95% CI)	Adjusted IRR (95% CI)	Unadjusted IRR (95% CI)	Adjusted IRR (95% CI)
Age	1.04 (1.01–1.07) ^*^	1.03 (0.99–1.06)	1.03 (1.01–1.07) ^*^	1.01 (0.98–1.05)
BMI	0.87 (0.83–0.93)^†^	0.88 (0.79–0.98) ^*^	0.84 (0.79–0.89) ^†^	0.92 (0.84–1.01)
Smoking (pack year)	1.00 (0.99–1.02)	1.00 (0.99–1.02)	1.01 (1.00-1.02) ^*^	1.01 (1.00-1.02) ^*^
CAT score	1.04 (1.02–1.08) ^*^	1.01 (0.97–1.06)	2.03 (1.10–3.75) ^*^	1.04 (1.00-1.08) ^*^
SGRQ total score	1.03 (1.01–1.04) ^†^	1.01 (0.99–1.03)	1.04 (1.03–1.05) ^†^	1.01 (0.99–1.03)
6MIN WT (m)	1.00 (0.99-1.00) ^*^	1.00 (1.00-1.01) ^*^	0.99 (0.99–0.99) ^†^	1.00 (0.99-1.00)
**Post-bronchodilator FEV**_**1**_ **-% of predicted value**	0.98 (0.97–0.99)^*^	0.98 (0.95–1.02)	0.97 (0.95–0.98) ^†^	0.99 (0.96–1.02)
**Post-bronchodilator ratio of FEV**_**1**_ **to FVC-%**	0.98 (0.96–0.99) ^*^	1.02 (0.97–1.02)	0.95 (0.93–0.97) ^†^	0.98 (0.94–1.03)
Previous year severe AE	2.09 (1.24–3.50) ^*^	1.49 (0.78–2.85)	2.53 (1.59–4.02) ^†^	1.21 (0.69–2.13)
Low cell viability	1.58 (1.01–2.50) ^*^	1.04 (0.59–1.84)	2.69 (1.66–4.35) ^†^	2.24 (1.30–3.86) ^†^

Abbreviations: IRR = Incidence rate ratio; CI = confidence interval, BMI = body mass index; CAT, COPD Assessment Test; SGRQ = St George’s Respiratory Questionnaire; 6MIN WT = 6 min walk test; AE = acute exacerbations; FVC = forced vital capacity; _FE1V_= forced expiratory volume in 1 s; DLCO = diffusing capacity of the lungs for carbon monoxide; TLC = total lung capacity; RV = residual volume.

*:*p* < 0.05 **†***p* < 0.005.

**Fig. 3:**
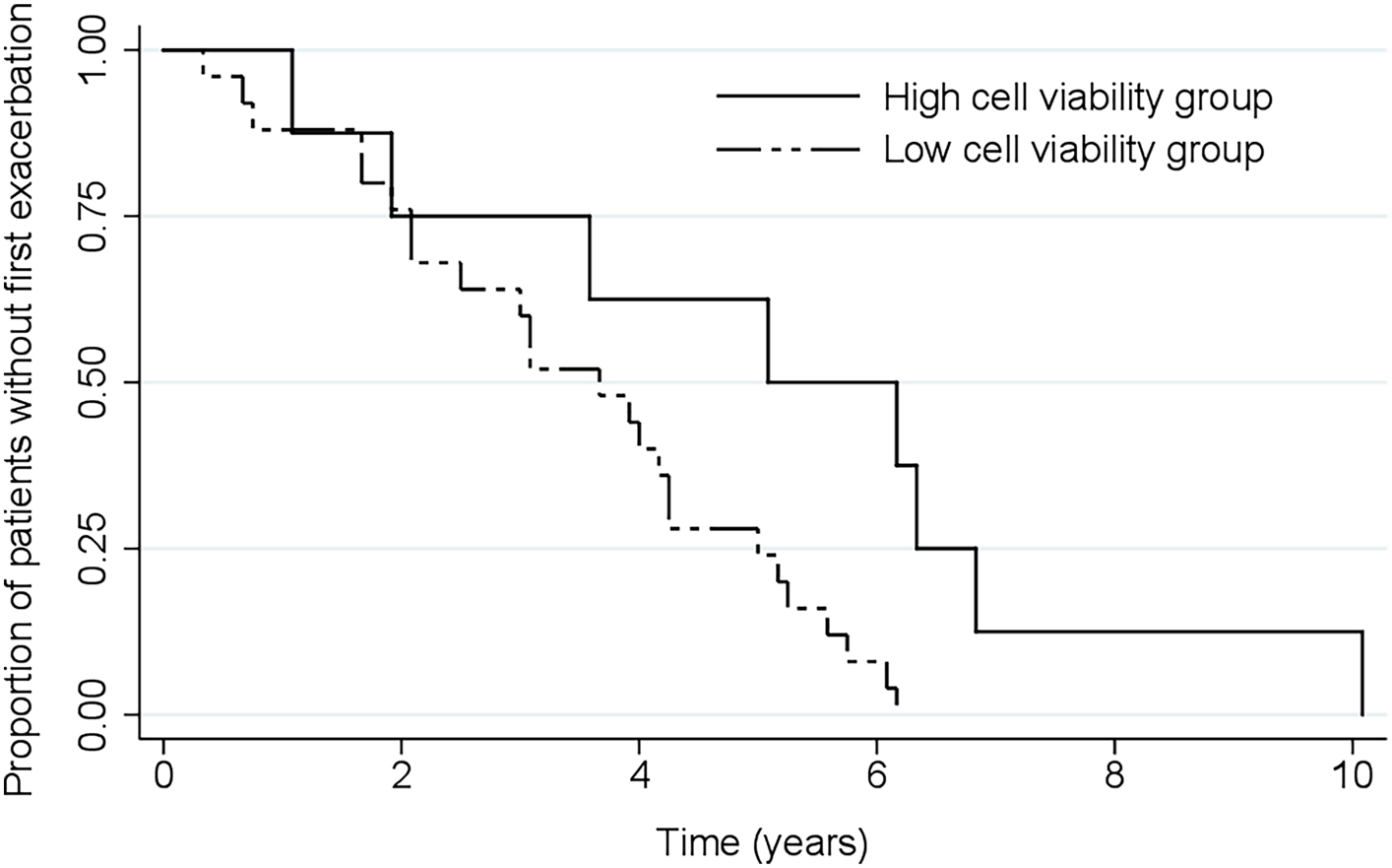
Kaplan-Meier curve of time to first overall COPD severe exacerbation leading to emergency room visit or hospitalization.

In addition, the low cell viability group had a significantly shorter time-to-first-severe exacerbation during follow-up than those in the high cell viability group (hazard ratio: 3.25, 95% CI: 1.17–8.99, *p* = 0.02) (Fig. 3).

### Mortality rate by cell viability

Death occurred in 21 (18.8%) patients during the follow- up period, of whom 70% died due to respiratory distress. Cell viability was associated with higher mortality, with an adjusted hazard ratio of 5.79 (95% CI: 1.38–24.23; *p* = 0.016) in the multivariable analysis (revised Supplementary Fig. 1). Older age, lower BMI, arrhythmia, heart failure, chronic kidney disease, higher baseline CAT and SGRQ scores, shorter 6-min walk test, and lower FVC were independently associated with increased all-cause mortality in the univariate analysis, while post-bronchodilator FEV1 (%) or the ratio of FEV1 to FVC (%) were not associated with mortality. Multivariate Cox-proportional hazard models showed that lower BMI, higher CAT score at baseline, and lower serum cell viability remained significant independent predictors of mortality (Table 3).

**Table 3:** Cox proportional hazard model analysis of risk factor for all-cause mortality.

	Univariate model		Multivariate model	
	HR (95% CI)	*P*	HR (95% CI)	*P*
Age	1.09 (1.03–1.16)	0.003	1.04 (0.98–1.12)	**0.225**
Sex (ref. male)	0.38 (0.52–2.72)	0.332	-	-
BMI	0.77 (0.69–0.87)	< 0.001	**0.69 (0.54–0.87)**	**0.002**
Eosinophil count	0.99 (1.00–1.00)	0.654	-	-
History of severe AE	2.94 (1.18–7.31)	0.02	**0.78 (0.19–3.15)**	**0.725**
Smoking (ref. never)				
Ex smoker	0.94 (0.12–7.21)	0.958	-	-
Current smoker	1.50 (0.18–12.52)	0.708	-	-
Smoking (PY)	1.01 (0.99–1.03)	0.206	-	-
Commorbidity				
Malignancy	1.13 (0.44–2.93)	0.366	-	-
Coronary disease	1.02 (0.34–3.04)	0.972	-	-
Arrhythmia	3.41 (1.14–10.16)	0.027	**6.52 (0.74–57.39)**	**0.091**
Heart failure	3.35 (1.12–10.01)	0.03	**2.40 (0.30-19.92)**	**0.416**
CKD	3.75 (1.26–11.18)	0.018	**1.37 (0.06–33.56)**	**0.846**
DM	2.68 (0.98–7.34)	0.055	**8.48 (0.58–123.90)**	**0.118**
CAT	1.09 (1.03–1.15)	0.001	**1.19 (1.06–1.34)**	**0.002**
SGRQ, total	1.03 (1.01–1.06)	0.016	**0.96 (0.92–1.01)**	**0.062**
6MIN WT	0.99 (0.99–0.99)	0.001	0.99 (0.99-1.00)	**0.836**
**Post-bronchodilator FEV**_1_-% of predicted value	0.99 (0.96–1.01)	0.278	-	-
**Post-bronchodilator ratio of FEV**_**1**_ **to FVC-%**	0.99 (0.95–1.02)	0.656	-	-
DLCO(%)	0.99 (0.97–1.01)	0.239	-	-
Low cell viability **(ref. high cell viability)**	3.47 (1.27–9.50)	0.015	**6.34 (1.46–27.46)**	**0.014**

Abbreviations. BMI = body mass index; AE = acute exacerbations; CKD = chronic kidney disease; DM = diabetes mellitus; CAT, COPD Assessment Test; SGRQ = St George’s Respiratory Questionnaire; 6MIN WT = 6 min walk test; FEV_1_ = forced expiratory volume in 1 s; DLCO = diffusing capacity of the lungs for carbon monoxide;.

## Discussion

This study is the first prospective cohort study to demonstrate that cell viability measured with a LDH cytotoxicity assay using serum from patients with COPD could be a good predictor of future AECOPD and mortality.

Phagocytosis of apoptotic cells (efferocytosis) is essential to maintain homeostasis of an organism to preserve health. Dysregulation of efferocytosis and accumulation of dead cells are involved in the pathogenesis of chronic inflammatory disease, including COPD^22^. Apoptotic cells produce “eat me” signals, and these signals are recognized by phagocytes under normal conditions. However, the activity of the phagocytosis signaling pathway is decreased in patients with COPD^20^^23^,. Therefore, an association between developing COPD and impaired clearance of dead cells has been established. Statin, azithromycin, and rosiglitazone enhance phagocytosis and apoptosis in COPD; lovastatin has been studied to restore efferocytotic function in vitro^24^^25^,. However, to the best of our knowledge, only one study reported the effect of efferocytosis dysregulation on COPD severity and exacerbations, showing that impaired macrophage efferocytosis of eosinophils measured using sputum was related to frequent COPD exacerbations^21^. However, it is unclear whether blood eosinophils could be a robust biomarker for predicting COPD exacerbation. In a recent study of 22,125 patients with COPD from 11 clinical trials, there was no significant association between blood eosinophils and exacerbation rate after adjusting for the previous-year exacerbation history^12^. This result corresponds with that of the present study, demonstrating that blood eosinophil count was not associated with exacerbation or mortality rates.

We found that the low cell viability group was significantly older, had a poorer quality of life by SGRQ score, a lower proportion of non-exacerbator phenotype, and a higher mortality rate than the high cell viability group. Furthermore, the low cell viability group demonstrated a trend for increased previous-year severe exacerbation history, higher CAT score and eosinophil counts, and shorter 6-min walk test duration; nonetheless, the between-group differences did not reach statistical significance. Furthermore, there were no differences in lung function or type of inhaler used between the two groups. Traditionally, the severity of COPD is classified using predicted FEV_1_ (%)^26^. Recently, a new classification system has emerged, called STAR (Staging of Airflow obstruction by ratio), which uses the FEV_1_/FVC ratio rather than predicted FEV_1_% to measure the severity of airflow obstruction. The COPDGene study and the Pittsburgh cohort revealed STAR had better discriminative ability for survival and patient symptoms than that of predicted FEV_1_ (%)^27^. However, this new classification system has not been studied in other races (such as Asians), albeit being less sensitive to race/ethnicity. In the present study, the FEV_1_/FVC ratio was not significantly associated with exacerbation incidence (adjusted IRR: 0.98, 95% CI: 0.94–1.03) and mortality (hazard ratio: 0.99, 95% CI: 0.97–1.02). In contrast to the STAR system, low cell viability was associated with increased unadjusted and adjusted IRRs of future severe AECOPD risk after adjustment for age, BMI, smoking history, CAT score, SGRQ score, 6-min walk test performance, predicted FEV_1_%, FEV_1_/FVC ratio, and previous exacerbation history within 1 year. Interestingly, a history of severe AECOPD in the previous year was associated with an increased unadjusted IRR for moderate and severe exacerbation, while a history of exacerbation was not found to be significantly associated with future moderate or severe exacerbation risk, after adjustment. Past history of exacerbations is associated with future exacerbation risk. This discrepancy could be explained by the limitation of recall bias associated with the history of exacerbation because longitudinal prospective cohort studies have reported that individuals with a similar exacerbation history had markedly varied trajectories of future exacerbation episodes^28,29^.

Regarding the type of inhaler used at baseline, the proportion of ICS + LABA (18.3%) and dual bronchodilator (15.7%) was similar. According to the GOLD 2017 guidelines, ICS + LABA is recommended for use in the group stratified by symptoms, lung function, and number of exacerbations or severity, regardless of the eosinophil count^30^. The use of these inhalers might have influenced the outcomes; however, there was no difference in inhaler type used between the low- and high-cell viability groups.

Additionally, our results showed that low cell viability was associated with a 6.34- fold increase in mortality, while a history of exacerbation in the previous year was not significantly associated with mortality after adjustment. This strong association should be interpreted with caution. Although BMI was included in the multivariate model as an indicator of nutritional status, it may not fully reflect general frailty or detailed aspects of nutritional conditions. Furthermore, socioeconomic status, which may also contribute to mortality risk, was not included in our analysis.

FEV1% and FEV1/FVC ratio were also not significantly associated with mortality, while low cell viability remained a significant predictor in the multivariate model. This finding contrasts with previous studies in which more severe airflow limitation was associated with an increased risk of exacerbation and mortality^31^. It suggests that cell viability may have prognostic value beyond traditional lung function markers. Importantly, this does not contradict the well-established prognostic role of FEV1 or FEV1/FVC ratio, but rather highlights the potential additive value of biological markers in predicting outcomes in patients with COPD. It is known that after the first severe exacerbation event requiring hospitalization, some patients remain stable without further exacerbation; however, after the second exacerbation event requiring hospitalization, there are frequent exacerbations with shorter intervals between them, and the mortality rate increases with each subsequent exacerbation^32^. The association between cell viability and survival (rather than the history of severe exacerbations) in patients with COPD could be explained by our finding that low cell viability was associated with more frequent exacerbations, leading to rapid deterioration of health status and increased mortality risk.

In addition, as we mentioned above it, defective clearance of dead cells can lead to the accumulation of necrotic cellular debris, which in turn promotes persistent systemic inflammation^20,33^. Chronic systemic inflammation is well-established pathogenic mechanism in COPD and is associated with increased mortality^34^. This mechanism could partly explain why low cell viability remained associated with high HR for mortality, even after adjustment for lung function and exacerbation history.

These findings suggest that low cell viability could be an indicator to identify patients who are more symptomatic, less able to exercise, more likely to have frequent exacerbation events, and have a higher mortality rate, irrespective of lung function and history of severe exacerbations in the previous year. However, clinical application of cell viability using LDH cytotoxicity assay warrants further consideration. Although this assay is relatively simple and cost-effective, further studies are needed to evaluate its reproducibility, standardization, and feasibility for automation in routine practice. Moreover, external validation in larger, multicenter cohorts is essential before recommending its widespread clinical use.

The present study has several strengths. First, it was the first study to evaluate the value of serum cell viability as a prognostic biomarker for future COPD exacerbation. Second, this was a prospective study with a substantial follow-up period, covering a median of 6.3 years. Third, we analyzed the various clinical parameters and lung function results that could be associated with COPD prognosis. Nonetheless, this study also has some limitations. First, never-smokers accounted for only 3.5% of our cohort; thus, the value of cell viability needs to be validated in patients with COPD without smoking history. Second, serum contains endogenous LDH, which can decrease the sensitivity of the LDH cytotoxicity assay or detection of cellular LDH. However, the serum was adequately diluted as described in methods, minimizing background interference. In addition, some samples were excluded due to excessive cell lysis or insufficient volume, which may introduce unquantified bias despite being based solely on technical limitations rather than clinical characteristics. Third, long term follow up period is both a strength and a limitation, as changes in covariates (e.g., treatment or newly developed diseases) might influence the assessment of association between cell viability and exacerbation or mortality. Fourth, although our cohort included a substantial proportion of patients with moderate to severe airflow obstruction (approximately 30% of patients had FEV1 < 50% predicted), those with very severe obstruction (FEV1 < 30%) were relatively few. This may limit the generalizability of our findings to patients with very severe COPD. Fifth, recall bias may have affected the accuracy of self-reported exacerbation history, potentially attenuating its association in the adjusted analysis. Sixth, the sample size was relatively small, which might have limited the statistical power particularly in subgroup analyses and multivariable models involving multiple covariates. However, we assessed multi-collinearity among covariates using variance inflation factors (VIFs), and found all values to be within acceptable ranges, supporting the stability of the multivariable analysis. Seventh, as this was an observational study, causality cannot be definitively established despite the prospective design. Last, the operational definition was used for the degree of cell death due to the absence of absolute reference value of OD for cell viability using serum. However, in cytotoxicity kits from various manufacturers, “low control” is typically defined as OD < 0.8, and “high control” as OD < 2.0, according to the manufacturer’s instructions^35^^36^,. Therefore, our median value of 0.737, which is close to the 0.8 threshold, would be not an unreasonable.

In conclusion, low cell viability measured by a serum LDH cytotoxicity assay was associated with further severe exacerbation and higher mortality in patients with COPD. However, in light of the limited sample size and the single-center design of our study, further research is needed to validate serum cell viability as a biomarker for COPD exacerbation and mortality risk in larger, independent cohorts.

## Methods

### Study design and setting

We conducted a prospective cohort study of patients with chronic airway disease at our tertiary care hospital, Seoul National Bundang Hospital (SNUBH), commencing in January 2012. Patients with asthma and COPD who visited the pulmonary clinic, provided written informed consent including agreement to blood sampling, and were prescribed an inhaler were enrolled from January 2012 to December 2016; follow-up was conducted in December 2023. Patients were excluded if they withdrew consent, had excessively high LDH levels that could confound background LDH effects, provided insufficient blood volume for sampling (< 3 mL), died within one year of follow-up, were lost to follow-up with unknown outcomes, or were diagnosed with asthma or interstitial lung disease (ILD). The institutional review board of SNUBH approved the study (IRB number: B-1108-134-004), and written informed consent was obtained from each patient. All methods were performed in accordance with the Declaration of Helsinki.

Both COPD and asthma were diagnosed by a pulmonologist according to the definitions of the GOLD and GINA guidelines, respectively, as shown below. Patients with COPD were defined as those with over 10 pack-years of smoking history, chronic respiratory symptom (cough, sputum, or dyspnea) and post-bronchodilator FE1V/FVC < 0.7 and FE1V < 80%^37^. Patients with Asthma were defined as those with a history of variable respiratory symptoms including wheeze, shortness of breath, chest tightness, or cough and positive bronchodilator reversibility test or broncho-provocation test^38^.

### Variables and Definitions.

At enrollment, the following patient data were collected: age, sex, blood eosinophil count, smoking history, prior history of exacerbations, COPD Assessment Test (CAT) score, St George’s Respiratory Questionnaire (SGRQ) score, 6-min walk test score, type of inhaler (inhaled corticosteroid (ICS), long-acting muscarinic antagonist (LAMA) or long-acting ß2 agonist (LABA)), pulmonary function test with post-bronchodilator, diffusing capacity of the lung for CO [DLCO], and total lung capacity. AECOPD was classified as moderate or severe. Moderate exacerbation was defined as worsened COPD symptoms (dyspnea, cough, or sputum) requiring treatment with systemic corticosteroids and/or antibiotics. Severe AECOPD was defined as worsening of COPD symptoms requiring emergency room visits or hospitalization.

### Blood sampling and cytotoxicity assay

Peripheral blood samples were obtained while patients were in a stable state of COPD. Blood sampling of patients who had experienced COPD exacerbation was conducted 1 month after the resolution of AECOPD. The blood samples from stable COPD patients were collected in serum separate tubes, ensuring a minimum volume of 3 mL, and stored appropriately for later analysis. For the cell viability test, measurements were performed in duplicate to assess the stability and reproducibility of the cytotoxicity results. The incubation period involved incubating the samples for 30 min under standard culture conditions. Following this, 50 µL of the supernatant was transferred for the measurement of LDH activity to minimize potential interference from serum-derived LDH. Therefore, the measured OD levels primarily reflect the cytotoxic effect of patient serum on the cells, rather than pre-existing LDH in the serum.

The Promega CytoTox 96^®^ Non-Radioactive Cytotoxicity Assay was used to determine cell viability. LDH is a stable enzyme in living cells and cannot penetrate the cell membrane under normal circumstances. However, LDH leaks out of the cell under circumstances of cell death or membrane damage. For this reason, the level of LDH can be used as an indicator of cell membrane integrity. Further, the method is well-established, fast, robust, and reproducible for assessing cell viability^39^^40^,The LDH cytotoxicity assay quantitatively measures LDH released from cells during lysis, resulting in the conversion of a tetrazolium salt into a red formazan product. The generation of this red formazan product is proportional to the amount of LDH released, and the intensity (absorbance at 490 nm) of color formed using a standard 96-well plate reader is proportional to the number of lysed cells]^41^^[,42^. The value of absorbance correlated to the number of damaged cells, and if the absorbance value is increased, it means that a large number of cells died. There is no set value or reference of OD, but it is generally recommended that OD does not exceed 2.0 in most cases, as values above this threshold may indicate a high background LDH effect. In addition, the variation of OD value should be less than 0.2 in repeated measurements, because if it is more than 0.2, it means large variation of absorbance. Therefore, it was necessary to determine an appropriate serum concentration that met these criteria. We diluted the serum with dextrose water at a ratio of 1:10. After confirming that the diluted serum satisfied the criteria, we proceeded with the experiment.

### Statistical analysis

Based on the optical density (OD) reading of the cytotoxicity assay, patients were divided into two groups based on the median value (OD 0.737), as there is no established cut-off value for this measurement. We operationally defined the group as low (OD > 0.737) and high (OD ≤ 0.737) cell viability groups. We utilized Student’s t-test or the Mann–Whitney U test to compare the continuous variables between the two groups (low cell viability group and high cell viability group), as appropriate. Fisher’s exact test or Pearson’s chi-square test was performed to analyze categorical variables. Annual rates of exacerbations were assessed using Poisson regression models adjusted for age, sex, exacerbation history, lung cancer, blood eosinophil count, and cell viability which was determined using the LDH cytotoxicity assay. A Cox proportional hazard model was used to estimate a hazard ratio for the time to a severe COPD exacerbation and mortality. To assess the potential for multicollinearity in the multivariable Cox proportional hazards model for mortality, VIFs were calculated for all covariates. All VIF values were below 10, with a mean VIF of 2.51, indicating no significant multi-collinearity (Supplementary Table 1). STATA 13 (STATA Corp LP., College Station, TX, USA) and R version 4.0.3 were used for statistical analyses. P-values < 0.05 were considered statistically significant.

## Supplementary Information

Below is the link to the electronic supplementary material.

Supplementary Material 1

Supplementary Material 2

Supplementary Material 3

Supplementary Material 4

## Data Availability

The data that support the findings of this study are available from the corresponding author upon reasonable request.

## Abbreviations

AECOPD: Acute exacerbation of COPD
BMI: Body mass index
CAT,COPD: Assessment Test
CI: Confidence interval
COPD: Chronic obstructive pulmonary disease
FEV_1_: Forced expiratory volume in 1s
FVC: Forced vital capacity
ICS: Inhaled corticosteroids
IRR: Incidence rate ratio
HR: Hazard ratio
KM: Kaplan–Meier
LABA: Long-acting β2 agonist
LDH: Lactate dehydrogenase
SGRQ: St George’s Respiratory Questionnaire
STAR: Staging of Airflow obstruction by ratio

## References

[1] Stolz, D. et al. Towards the elimination of chronic obstructive pulmonary disease: a lancet commission. *Lancet* **400**, 921–972. 10.1016/S0140-6736(22)01273-9 (2022). 36075255 10.1016/S0140-6736(22)01273-9 PMC11260396

[2] Halpern, M. T., Stanford, R. & Borker, R. The burden of COPD in the USA: results from the confronting COPD survey. *Respir. Med.* **97**, S81–S89 (2003). 12647946 10.1016/s0954-6111(03)80028-8

[3] Waeijen-Smit, K. et al. All-cause admissions following a first ever exacerbation-related hospitalisation in COPD. *ERJ Open. Res.* **9**, 00217–02022. 10.1183/23120541.00217-2022 (2023). 36605904 10.1183/23120541.00217-2022 PMC9808537

[4] Jo, Y. S. et al. Development of a daily predictive model for the exacerbation of chronic obstructive pulmonary disease. *Sci. Rep.* **13**, 18669. 10.1038/s41598-023-45835-4 (2023). 37907619 10.1038/s41598-023-45835-4 PMC10618439

[5] Guerra, B., Gaveikaite, V., Bianchi, C. & Puhan, M. A. Prediction models for exacerbations in patients with COPD. *Eur. Respir Rev.* **26**10.1183/16000617.0061-2016 (2017). 10.1183/16000617.0061-2016 PMC9489020 28096287

[6] Yao, Y., Zhou, J., Diao, X. & Wang, S. Association between tumor necrosis factor-α and chronic obstructive pulmonary disease: a systematic review and meta-analysis. *Ther. Adv. Respir Dis.* **13**, 1753466619866096. 10.1177/1753466619866096 (2019). 31390957 10.1177/1753466619866096 PMC6688146

[7] Perera, W. R. et al. Inflammatory changes, recovery and recurrence at COPD exacerbation. *ERJ* **29**, 527–534 (2007). 10.1183/09031936.00092506 17107990

[8] Chen, Y. W. R., Leung, J. M. & Sin, D. D. A systematic review of diagnostic biomarkers of COPD exacerbation. *PloS One*. **11**, e0158843 (2016). 27434033 10.1371/journal.pone.0158843 PMC4951145

[9] Fermont, J. M. et al. Biomarkers and clinical outcomes in COPD: a systematic review and meta-analysis. *Thorax* **74**, 439–446 (2019). 30617161 10.1136/thoraxjnl-2018-211855 PMC6484697

[10] Singh, D. Blood eosinophil counts in chronic obstructive pulmonary disease: A biomarker of inhaled corticosteroid effects. *Tuberc Respir Dis (Seoul)*. **83**, 185–194. 10.4046/trd.2020.0026 (2020). 32578413 10.4046/trd.2020.0026 PMC7362755

[11] Ashdown, H. F. et al. Blood eosinophils to guide inhaled maintenance therapy in a primary care COPD population. *ERJ Open. Res.* **8**, 00606–02021. 10.1183/23120541.00606-2021 (2022). 35141324 10.1183/23120541.00606-2021 PMC8819252

[12] Singh, D. et al. Blood eosinophils as a biomarker of future COPD exacerbation risk: pooled data from 11 clinical trials. *Resp. Res.* **21**, 240. 10.1186/s12931-020-01482-1 (2020). 10.1186/s12931-020-01482-1 PMC7499955 32943047

[13] Vedel-Krogh, S., Nielsen, S. F., Lange, P., Vestbo, J. & Nordestgaard, B. G. Blood eosinophils and exacerbations in chronic obstructive pulmonary disease. The Copenhagen general population study. *Am. J. Respir Crit. Care Med.* **193**, 965–974. 10.1164/rccm.201509-1869OC (2016). 26641631 10.1164/rccm.201509-1869OC

[14] Kerkhof, M. et al. Blood eosinophil count and exacerbation risk in patients with COPD. *ERJ* **50**, 1700761. 10.1183/13993003.00761-2017 (2017). 10.1183/13993003.00761-2017 28729477

[15] Kasahara, Y. et al. Endothelial cell death and decreased expression of vascular endothelial growth factor and vascular endothelial growth factor receptor 2 in emphysema. *Am. J. Respir Crit. Care Med.* **163**, 737–744. 10.1164/ajrccm.163.3.2002117 (2001). 11254533 10.1164/ajrccm.163.3.2002117

[16] Hodge, S., Hodge, G., Holmes, M. & Reynolds, P. N. Increased airway epithelial and T-cell apoptosis in COPD remains despite smoking cessation. *ERJ* **25**, 447–454. 10.1183/09031936.05.00077604 (2005). 10.1183/09031936.05.00077604 15738287

[17] Henson, P. M., Vandivier, R. W. & Douglas, I. S. Cell death, remodeling, and repair in chronic obstructive pulmonary disease? *Proc. Am. Thorac. Soc.* **3**, 713–717. 10.1513/pats.200605-104SF (2006). 17065379 10.1513/pats.200605-104SF PMC2647658

[18] Asare, P. F. et al. Inhibition of LC3-associated phagocytosis in COPD and in response to cigarette smoke. *Ther. Adv. Respir Dis.* **15**10.1177/17534666211039769 (2021). 10.1177/17534666211039769 PMC8647217 34852704

[19] Gerlach, B. D. et al. Efferocytosis induces macrophage proliferation to help resolve tissue injury. *Cell. Metab.* **33**, 2445–2463e8. 10.1016/j.cmet.2021.10.015 (2021). 34784501 10.1016/j.cmet.2021.10.015 PMC8665147

[20] Zheng, W. et al. Efferocytosis and respiratory disease. *Int. J. Mol. Sci.* **24**, 14871. 10.3390/ijms241914871 (2023). 37834319 10.3390/ijms241914871 PMC10573909

[21] Eltboli, O. et al. COPD exacerbation severity and frequency is associated with impaired macrophage efferocytosis of eosinophils. *BMC Pulm Med.* **14**, 112. 10.1186/1471-2466-14-112 (2014). 25007795 10.1186/1471-2466-14-112 PMC4115214

[22] Doran, A. C., Yurdagul, A. & Tabas, I. Efferocytosis in health and disease. *Nat. Rev. Immunol.* **20**, 254–267. 10.1038/s41577-019-0240-6 (2020). 31822793 10.1038/s41577-019-0240-6 PMC7667664

[23] Wang, Y. et al. Cigarette smoke attenuates phagocytic ability of macrophages through down-regulating milk fat globule-EGF factor 8 (MFG-E8) expressions. *Sci. Rep.* **7**, 42642. 10.1038/srep42642 (2017). 28195210 10.1038/srep42642 PMC5307389

[24] Walsh, G. M. Defective apoptotic cell clearance in asthma and COPD – a new drug target for statins? *Trends Pharmacol. Sci.* **29**, 6–11. 10.1016/j.tips.2007.11.002 (2008). 18054798 10.1016/j.tips.2007.11.002

[25] Morimoto, K. et al. Lovastatin enhances clearance of apoptotic cells (efferocytosis) with implications for chronic obstructive pulmonary disease. *J. Immun.* **176**, 7657–7665 (2006). 16751413 10.4049/jimmunol.176.12.7657

[26] Agustí, A. et al. Global initiative for chronic obstructive lung disease 2023 report: GOLD executive summary. *Am. J. Respir Crit. Care Med.* **207**, 819–837 (2023). 36856433 10.1164/rccm.202301-0106PP PMC10111975

[27] Bhatt, S. P. et al. FEV(1)/FVC severity stages for chronic obstructive pulmonary disease. *Am. J. Respir Crit. Care Med.* **208**, 676–684. 10.1164/rccm.202303-0450OC (2023). 37339502 10.1164/rccm.202303-0450OC PMC10515563

[28] Han, M. K. et al. Frequency of exacerbations in patients with chronic obstructive pulmonary disease: an analysis of the SPIROMICS cohort. *Lancet Respir Med.* **5**, 619–626. 10.1016/S2213-2600(17)30207-2 (2017). 28668356 10.1016/S2213-2600(17)30207-2 PMC5558856

[29] Chaudhary, M. F. A. et al. Predicting severe chronic obstructive pulmonary disease exacerbations using quantitative CT: a retrospective model development and external validation study. *Lancet Digit. Health*. **5**, e83–e92. 10.1016/s2589-7500(22)00232-1 (2023). 36707189 10.1016/S2589-7500(22)00232-1 PMC9896720

[30] Rodriguez-Roisin, R., Rabe, K. F., Vestbo, J., Vogelmeier, C. & Agustí, A. Global initiative for chronic obstructive lung disease (GOLD) 20th anniversary: a brief history of time. *ERJ* **50**10.1183/13993003.00671-2017 (2017). 10.1183/13993003.00671-2017 28679615

[31] Marott, J. L. et al. Predicting exacerbations in COPD in the Danish general population. *Respir Med.* **224**, 107557. 10.1016/j.rmed.2024.107557 (2024). 38355020 10.1016/j.rmed.2024.107557

[32] Ware, S. A. et al. Cell-free DNA levels associate with COPD exacerbations and mortality. *Respir Res.* **25**10.1186/s12931-023-02658-1 (2024). 10.1186/s12931-023-02658-1 PMC10797855 38238743

[33] Dhawan, U. K., Singhal, A. & Subramanian, M. Dead cell and debris clearance in the atherosclerotic plaque: mechanisms and therapeutic opportunities to promote inflammation resolution. *Pharmacol. Res.* **170**, 105699. 10.1016/j.phrs.2021.105699 (2021). 34087352 10.1016/j.phrs.2021.105699

[34] Barnes, P. J. Inflammatory mechanisms in patients with chronic obstructive pulmonary disease. *J. Allergy Clin. Immunol.* **138**, 16–27. 10.1016/j.jaci.2016.05.011 (2016). 27373322 10.1016/j.jaci.2016.05.011

[35] Elabscience Cytotoxicity Assay Kit Manual. [PDF]. Available at: https://file.elabscience.com/biochemical_kits/ELISA-Cytotoxicity-Manual.pdf (Accessed: 28 March 2025).

[36] BRIC. Adjust the reaction time with appropriate absorbance values… PDF]. Available at: https://www.ibric.org/biomarket/sales-event (Accessed: 28 March 2025).

[37] Global Initiative for Chronic Obstructive Lung Disease (GOLD). Global strategy for the diagnosis, management, and prevention of COPD (2016). https://goldcopd.org/10.3760/cma.j.issn.0376-2491.2016.34.001 27667101

[38] Global Initiative for Asthma (GINA). Global strategy for asthma management and prevention (2016). https://www.ginasthma.org

[39] Hiebl, B., Engin, S., Gemeinhardt, O., Niehues, S. & Jung, F. Impact of serum in cell culture media on in vitro lactate dehydrogenase (LDH) release determination. *J. Cell. Biotechnol.* **3**, 9–13. 10.3233/JCB-179002 (2017).

[40] Vega-Avila, E. & Pugsley, M. K. An overview of colorimetric assay methods used to assess survival or proliferation of mammalian cells. *Proc. West. Pharmacol. Soc.* **54**, 10–14 (2011). 22423572

[41] Riss, T., Niles, A., Moravec, R., Karassina, N. & Vidugiriene, J. in (eds Assay, G., Manual, S., Markossian et al.) (Eli Lilly & Company and the National Center for Advancing Translational Sciences, (2004).

[42] Corporation, P. Total cell quantitation using the CytoTox 96™ Non-Radioactive Cytotoxicity Assay. 2800 Woods Hollow Road. Madison, WI USA, (1994).

