# Supplementary figures and images for: Cell viability measured by cytotoxicity assay as a biomarker of chronic obstructive pulmonary disease exacerbation: a prospective cohort study
Source: Sci Rep. 2025 Aug 7;15:28843. doi: 10.1038/s41598-025-14536-5 (PMC12329023; doi:10.1038/s41598-025-14536-5)

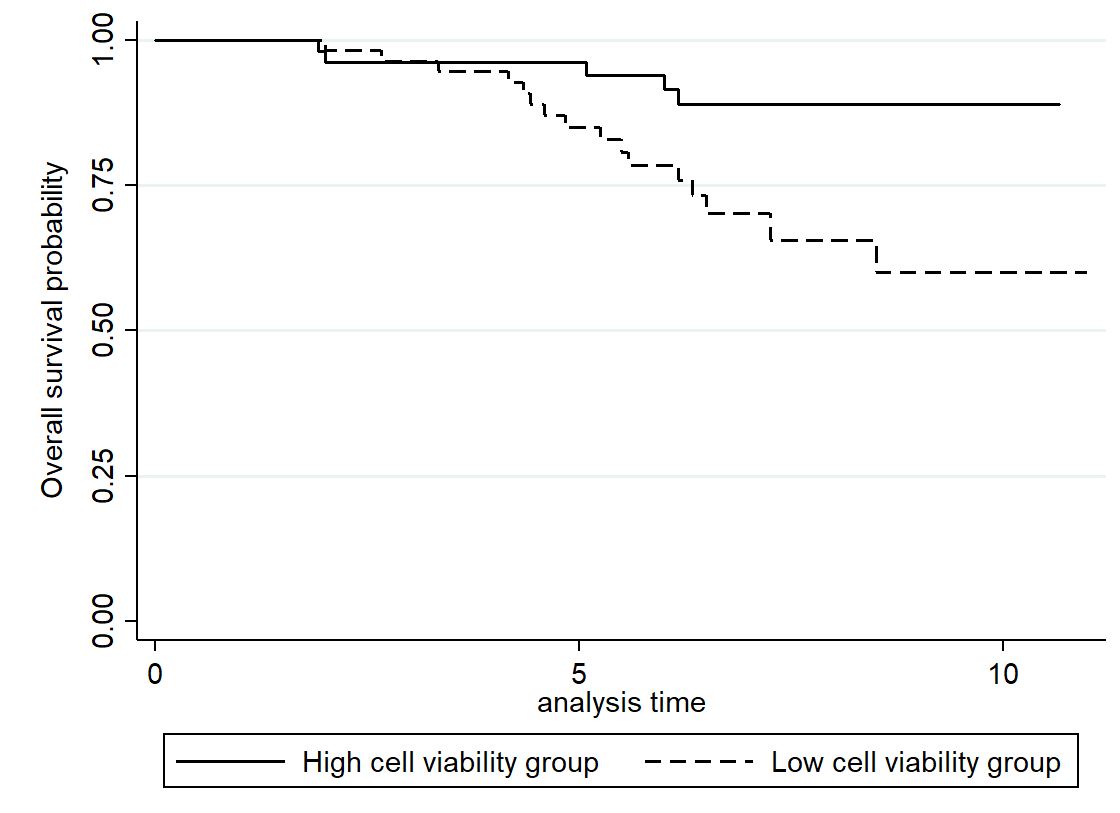

Supplement: Supplementary file 1 — Supplementary Material 1 [file 41598_2025_14536_MOESM1_ESM.jpg]

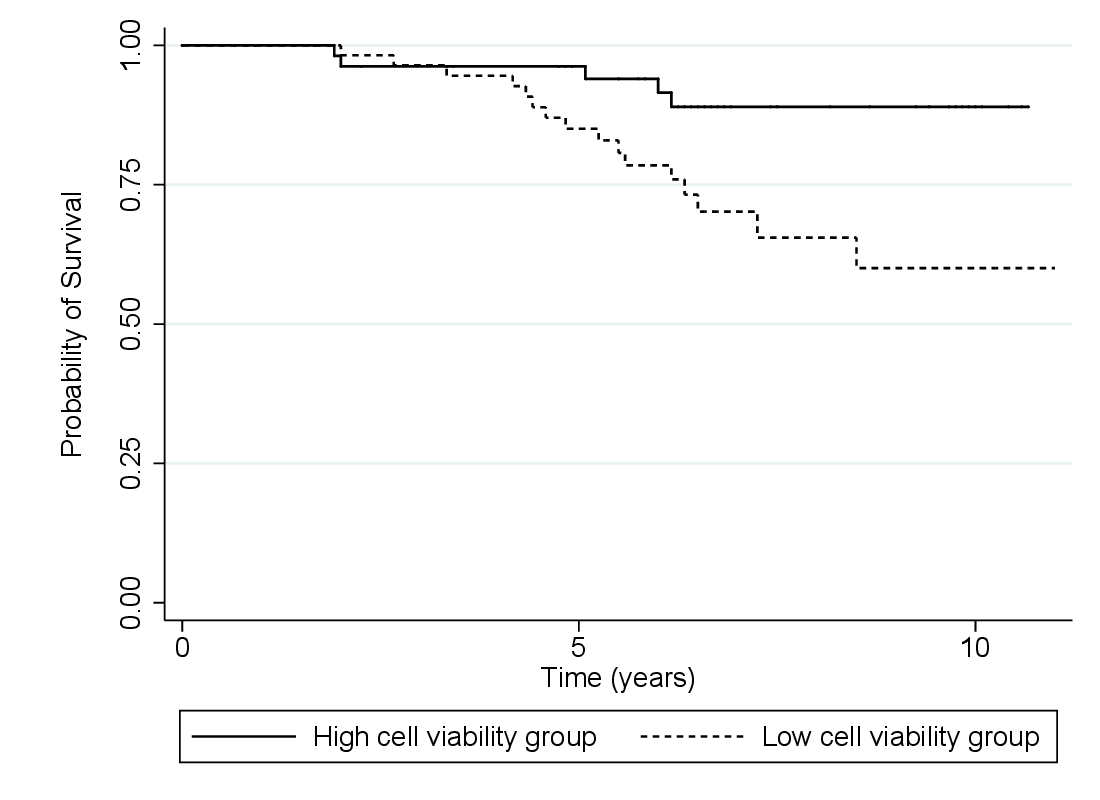

Supplement: Supplementary file 2 — Supplementary Material 2 [file 41598_2025_14536_MOESM2_ESM.tiff]
