# Supplementary material for: Cell viability measured by cytotoxicity assay as a biomarker of chronic obstructive pulmonary disease exacerbation: a prospective cohort study
Source: Sci Rep. 2025 Aug 7;15:28843. doi: 10.1038/s41598-025-14536-5 (PMC12329023; doi:10.1038/s41598-025-14536-5)
Supplement: Supplementary file 3 — Supplementary Material 3 [file 41598_2025_14536_MOESM3_ESM.docx]

**Supplementary Table 1. Colinearity for motality adjusted variables**

| **Variable** | **VIF** | **SQRT VIF** | **Tolerance** | **R-Squared** |
| --- | --- | --- | --- | --- |
| Age | 1.74 | 1.32 | 0.5756 | 0.4244 |
| Sex (ref. male) | 1.39 | 1.18 | 0.7184 | 0.2816 |
| BMI | 2.03 | 1.42 | 0.4933 | 0.5067 |
| Eosinophil count | 1.22 | 1.1 | 0.8224 | 0.1776 |
| History of severe AE | 1.5 | 9 1.26 | 0.6278 | 0.3722 |
| Smoking (ref. never) | 1.75 | 1.32 | 0.5699 | 0.4301 |
| Smoking (PY) | 1.41 | 1.19 | 0.7073 | 0.2927 |
| Malignancy | 1.25 | 1.12 | 0.7985 | 0.2015 |
| Coronary disease | 1.24 | 1.12 | 0.8033 | 0.1967 |
| Arrhythmia | 2.07 | 1.44 | 0.4827 | 0.5173 |
| Heart failure | 1.46 | 1.21 | 0.6859 | 0.3141 |
| CKD | 1.93 | 1.39 | 0.5188 | 0.4812 |
| DM | 1.79 | 1.34 | 0.5596 | 0.4404 |
| CAT | 1.67 | 1.29 | 0.5994 | 0.4006 |
| SGRQ, total | 2.13 | 1.46 | 0.4692 | 0.5308 |
| 6MIN WT | 1.79 | 1.34 | 0.5591 | 0.4409 |
| Post-bronchodilator FEV1-% of predicted value | 7.38 | 2.72 | 0.1354 | 0.8646 |
| Post-bronchodilator ratio of FEV1 to FVC-% | 7.44 | 2.73 | 0.1344 | 0.8656 |
| DLCO(%) | 2.13 | 1.46 | 0.4689 | 0.5311 |
| **Mean VIF** | **2.51** |  |  |  |
